# Factorial Validity of the Work Ability Index Among Employees in Germany

**DOI:** 10.1007/s10926-018-9803-9

**Published:** 2018-08-01

**Authors:** Marion Freyer, Maren Formazin, Uwe Rose

**Affiliations:** Federal Institute for Occupational Safety and Health, Nöldnerstr. 40-42, 10317 Berlin, Germany

**Keywords:** Work ability, Random sample, Survey, CFA, WAI

## Abstract

*Purpose* The Work Ability Index (WAI) is a routinely applied instrument for the assessment of work ability. It is a single score index, based on the implicit assumption of a single factor underlying the construct of work ability. The few studies with a focus on the WAI’s factor structure are mainly based on non-representative samples. The objective of this study was to examine the factor structure of the WAI within a representative sample of employees working in Germany, applying analysis procedures that consider the metric of the variables. *Methods* Analyses are based on a nationwide representative sample of employees aged 31–60 years from the “Study on Mental Health at Work” (German: S-MGA). Responses from n = 3968 participants were used in confirmatory factor analyses comparing competing models of the structure underlying the WAI. *Results* The results of the analyses suggest that the intercorrelations between the indicators of the WAI are explained better by a model with two correlated factors than by a simple one-factor structure. A model solely allowing a single loading for each indicator fits the data well and allows for an easy interpretation of the two underlying factors. *Conclusions* There are two correlated factors underlying the WAI: one refers to “subjective work ability and resources”, the other one can be considered a “health related factor”.

## Introduction

In the last decades, the concept of work ability and its operationalization have become a popular topic in occupational medicine [1, 2]. Work ability is defined as a person’s potential to manage his/her work tasks, taking into account the person’s health, working conditions and mental resources [3, 4]. The so-called “balance model of work ability” is the basic model of work ability [4]. It considers both, the demands within a person—which are a consequence of external workloads—and the individual’s resources to handle these demands [5]. A good balance leads to health, work ability and occupational well-being, while imbalance can be followed by overload and work-related illnesses [4]. According to this model, work ability depends on multiple factors and is described as the sum of factors that enable a person in a given situation to successfully master a given task [6].

The Work Ability Index (WAI) as a multidimensional diagnostic tool has been developed in Finland because no suitable instrument for the subjective assessment of the ability to work had been available [5, 7]. It has been used for studies aimed at investigating age related developments of work ability within occupational groups and work ability’s associations with type of pension and mortality [8]. Work ability was operationalized as the current and future work ability in relation to physical and mental work demands, health and individual resources. The WAI is calculated as an unweighted sum score over the WAI’s seven dimensions WAI1 to WAI7. In the following, the term ‘indicators’ will be used to refer to these seven dimensions (as named in previous literature on the WAI).

Compared to other questionnaires referring to functional capacity, the WAI assesses the ability to work directly. In contrast, questionnaires like the Norwegian Function Assessment [9] or the Short-Form-36 Health Survey [10] assess global aspects of functioning and are not restricted to the work domain.

The WAI’s validity has been shown in relation to external criteria such as clinical assessments, mortality and receiving disability pension within a period of 11 years [11, 12]. The results indicate that the predictive power of the WAI is mainly attributable to those indicators not explicitly related to health, namely WAI6, WAI2, WAI4 and WAI1 (in that order) [1]. Later studies replicated these results [13, 14]. Based on these results, one may wonder whether there is more than one factor underlying the WAI, especially when considering the high predictive ability of WAI6 (own prognosis of work ability 2 years from now) for receiving disability pension. Recent studies on determinants of return to work among employees with common mental disorders point in a similar direction [15, 16]: positive expectations regarding return to work—which show an overlap to one’s own prognosis of work ability—were a good predictor of successful return to work as was a higher WAI.

The calculation of the index as an unweighted sum score relies on the tacit assumption of unidimensionality, although—as has been described above—work ability is by definition multidimensional. This assumption also underlies the calculation of Cronbach’s alpha as a measure of internal consistency for the WAI which has been reported to range from α = 0.54 to α = 0.83 in different samples [17–22].

Analyses of the WAI’s underlying structure—applying different methods of sampling, different statistics and using the questionnaire in different languages and different cultures—have led to heterogeneous results, rendering the WAI’s interpretation difficult: a study in the context of the Second German Sociomedical Panel of Employees with 1036 employees aged 45 and above supported the assumption of one underlying factor [18]. However, confirmatory factor analyses (CFA) based on a German sample of several non-representative occupational groups revealed better fit of a two-factor compared to a one-factor model [20]. The first factor with loadings of the indicators WAI1, WAI2 und WAI7 was interpreted as reflecting the subjective assessment of work ability and resources while the second factor with loadings of indicators WAI3 und WAI5 was interpreted as a health-related factor. The indicators WAI4 und WAI6 showed an inconsistent pattern for each of the subgroups considered in this study. The interpretation of the study’s results is impeded by its ad hoc sample consisting of 324 female office workers, female nursery teachers as well as male and female teachers [20, 23].

In a study—not restricted to Germany but to one profession—with about 40,000 nurses from different European countries, country-specific differences in the factor structure of the WAI were found [22]. Based on principal components analyses, a one-factor structure was found for Germany and Finland whereas a concordant two-factor structure was found for the remaining countries. The authors of the European nurses’ study interpreted the factor underlying indicator WAI1, WAI2, WAI6 and WAI7 as subjective and the factor underlying indicators WAI3, WAI4 and WAI5 as objective components of work ability [22].

Other studies with translated versions of the WAI have found heterogeneous factor structures as well: In a Greek sample [24], two factors in accordance with Martus et al. [20] were established, while a three factor structure was found for an Iranian, a Brazilian and an Argentinean sample [17, 19, 21].

Furthermore, the heterogeneity of the results for the factor structure of the WAI could lie in the different occupational groups. As mentioned, Martus et al. [20, 23] examined an ad hoc sample of various working populations while other studies analysed occupations such as nurses and healthcare workers [17, 18, 21], blue and white collar workers of the shipyard industry [24] and workers of an electrical utility company [19]. This renders the interpretation and generalization of results for other professions difficult.

Consequently, the results on the WAI’s factor structure as presented above do not allow for a final conclusion because these results are based on different versions of the questionnaire due to translations, applied in different samples (regarding age, culture and occupation), analysed with different statistical methods.

Another aspect that has not been sufficiently considered in earlier studies is the scale level of the WAI’s indicators. So far, a metric scale level has been assumed in almost all studies: factor analyses were based on the covariance-matrix with product-moment correlations. However, not all WAI indicators can be considered as metric but rather as ordinal. For these ordinal variables, the polychoric correlation is the method of choice to avoid underestimation of the true relationships between the variables [25, 26].

The objective of the present study is to examine the factor structure of the WAI applying CFA and considering the indicators’ scale level. Analyses shall be based on a representative sample of employees in Germany, considering a lot of professional groups, to avoid possible bias due to the method of sampling. Based on results of Martus et al. [20] and Radkiewicz and Widerszal-Bazyl [22], it is assumed that a one-factor structure of the WAI will not be confirmed. Rather, it is expected that a two-factor model with indicators loading on only one factor at a time will show better model fit. The two factors will represent the subjective work ability and resources on the one hand and a health related factor on the other hand.

## Methods

### Populations

Data for this study stems from the baseline survey of ‘The Study on Mental Health at Work’ (German: S-MGA), a nationwide representative panel study with data collected in 2011/2012 (baseline) and in 2017 (follow up) by the infas Institute of Applied Social Sciences. The data are subject to national data protection laws and restrictions on data usage were imposed by the Institute for Employment Research (IAB) to ensure data privacy of the study participants. The cohort profile gives a comprehensive description of the study design, sampling procedure and data collection [27]. The sampling is based on all employees in Germany who were subject to social security contributions at the time of sampling with an age range from 31 to 60 years; self-employed, freelancers and civil servants are not part of the sample. In the first step of a two-stage area cluster sampling, from all municipalities in Germany, a random sample of 206 municipalities—proportionally stratified by region and population size—was selected. In the second step, a random sample of 13,590 addresses was drawn from these municipalities to obtain the aspired number of 4500 interviews. In the end, 4511 interviews were conducted by 243 trained interviewers using a computer-assisted personal interview. The participants gave their informed consent to the study and received an incentive of 10 Euros. The study was approved by the Research & Development Council of the Federal Institute for Occupational Safety and Health in Germany.

Socio-demographic and regional characteristics were used for comparing population, gross sample and respondents. The results indicate no major deviations, thus the quality of the sampling process can be considered as high [27].

### Work Ability Index (WAI)

For the present study, the German version of the WAI was used, which is based on a translation of the second revised English version [7, 28]. For the development of the WAI, 10 items from a collection of items about work, ability to work, health and psychological reserves were selected, taking into account the item’s correlations and a cross-classification [8]. These 10 items form the basis for the WAI’s seven indicators: while five indicators (WAI1, WAI3, WAI4, WAI5 and WAI6) are made up of one item each, one indicator (WAI2) is formed by two individual items which are weighted according to the instructions to calculate the WAI [7] and another indicator (WAI7) uses a transformation of an unweighted sum score over three items [7]. While the original WAI contains a list of 51 diseases for WAI3, we have used a version with a shorter list of 14 disease groups [29]. The WAI’s indicators are heterogeneous with different response formats and different scale levels (see also Table 1 second column): WAI1 ‘subjective estimation of current work ability compared with lifetime best’ with 1 item and a 11-point response scale (0–10 points); WAI2 ‘subjective work ability in relation to job demands’ with 2 items with a 5-point response scale for 1–5 points (the points are integrated into a formula and weighted according to the specified work requirements); WAI3 ‘number of current diseases diagnosed by a physician’ with a list of 14 disease groups (depending on the number of disease groups marked leading to 1, 3, 5 or 7 points); WAI4 ‘subjective estimation of work impairment due to diseases’ with 1 item and a 6-point response scale (1–6 points); WAI5 ‘sick leave during past year’ with 1 item and a 5-point response scale (1–5 points); WAI6 ‘own prognosis of work ability 2 years from now’ with 1 item and a 3-point response scale (1, 4 or 7 points) and WAI7 ‘mental resources’ with 3 items and a 5-point response scale with 0–4 points each, summed to a score which in turn is transformed to 1–4 points.

**Table 1:** Means, standard deviations and polychoric intercorrelations for the items of the Work Ability Index

Item		*M*	*SD*	1	2	3	4	5	6	7	8	9
1	Age group	–	–	–								
2	Sex	–	–	0.06 (0.02)	–							
3	WAI total	40.22	6.20	− 0.18 (0.02)	− 0.02 (0.02)							
4	WAI1: current work ability (1 item with 0–10 points)	8.02	1.78	− 0.13 (0.02)	0.02 (0.02)	0.76 (0.01)						
5	WAI2: work ability in relation to job demands (2 items with a sum of 2–10 points)	8.41	1.37	− 0.19 (0.02)	0.01 (0.02)	0.73 (0.01)	0.59 (0.01)					
6	WAI3: number of current diseases (last 12 months) (14 disease groups: 1, 3, 5 or 7 points)	4.84	1.95	− 0.13 (0.02)	− 0.13 (0.02)	0.67 (0.01)	0.29 (0.02)	0.29 (0.02)				
7	WAI4: estimated work impairment due to diseases (1 item with 1–6 points)	5.32	0.95	− 0.16 (0.02)	− 0.00 (0.02)	0.73 (0.01)	0.52 (0.01)	0.51 (0.01)	0.49 (0.01)			
8	WAI5: sick leave (last 12 months) (1 item with 1–5 points)	3.98	1.01	− 0.02 (0.02)	− 0.02 (0.02)	0.54 (0.01)	0.29 (0.02)	0.26 (0.02)	0.39 (0.02)	0.39 (0.02)		
9	WAI6: estimation of own work ability 2 years from now (1 item with 1, 4 or 7 points)	6.20	1.63	− 0.19 (0.02)	− 0.01 (0.03)	0.71 (0.01)	0.37 (0.02)	0.47 (0.02)	0.30 (0.02)	0.51 (0.02)	0.25 (0.02)	
10	WAI7: mental resources (3 items with a sum of 1–4 points)	3.36	0.70	− 0.06 (0.02)	0.13 (0.02)	0.59 (0.01)	0.43 (0.01)	0.48 (0.01)	0.26 (0.02)	0.36 (0.02)	0.22 (0.02)	0.46 (0.02)

*N* = 3968. *M* = mean, *SD* = standard-deviation, standard-error in brackets

Sex: 1 = male, 2 = female

Age group: 1 = 31–40 years, 2 = 41–50 years, 3 = 51–60 years

The range of the traditional WAI, calculated as an unweighted sum score over the seven indicators WAI1 to WAI7 based on the specifications in the manual [7], can vary between 7 and 49 points, with higher values indicating better work ability.

### Confirmatory Factor Analyses

The WAI’s factor structure was investigated by applying CFA using Mplus (version 7.4; Muthén & Muthén, Los Angeles, CA) based on the polychoric correlation matrix [30]. In absence of a multivariate normal distribution and to take into account the ordinal metric of the indicators WAI3 to WAI7, a robust mean- and variance-adjusted weighted least squares estimation procedure (WLSMV) was used for CFA. Previous studies found a superiority of the WLSMV estimation compared to the maximum likelihood (ML) estimation, taking into account the sample size, number of categories, and the nonnormal latent distribution [31–33]. Within WLSMV estimation, Mplus uses a pairwise deletion approach for handling missing data as the default. For reliability analyses in IBM SPSS Statistics (version 23.0; IBM Corp., Armonk, NY), subjects with missing data were excluded by listwise deletion leading to an exclusion rate of < 5% [34].

**Fig. 1:**
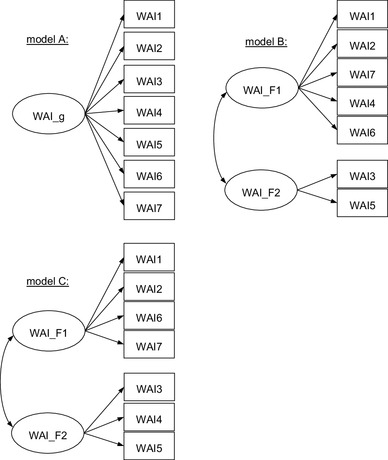
Tested models

Three models (Fig. 1) were specified based on previous analyses [20, 22]. Model A represents the assumption of unidimensionality of the WAI with only one factor WAI_g as a latent variable underlying the responses on all indicators WAI1 to WAI7. In model B—inspired by Martus et al. [20]—the indicators WAI1, WAI2, WAI4, WAI6 and WAI7 load on one factor WAI_F1 and indicators WAI3 and WAI5 on a separate, correlated factor WAI_F2. In this model, the first factor represents the subjectively assessed ability to work, work impairment as well as the individual resources. The second factor represents the number of diagnosed diseases and sick leave in the past year. Since Martus et al. [20] also present an additional model with double-loadings for WAI4 and WAI6, the former with a higher loading on the second factor and the latter loading higher on the first factor, a third model C was established. In this model, the indicators WAI1, WAI2, WAI6 and WAI7 load on one factor and the indicators WAI3, WAI4 and WAI5 on a second factor. It is assumed that the first factor represents the subjective perception of work ability and resources while the second factor comprises health-related conditions.

Factor loadings were estimated freely by fixing the factor variance to 1. Several indices were used to evaluate model fit: the χ^2^-test is reported as a standard index for the evaluation of different models despite its dependency on the sample size as well as its sensitivity to violations of the multivariate normal distribution [35]. In addition to the χ^2^-test, the root mean square error of approximation (RMSEA), the comparative fit index (CFI) and the Tucker–Lewis index (TLI) were also considered. The RMSEA as well as the CFI and the TLI are determined based on the χ^2^ by considering the sample size and/or the degrees of freedom. With reference to the discussion of guidelines [35, 36], the following rules for the evaluation of fit indices were used: a RMSEA ≤ 0.05 indicates good and a RMSEA > 0.05 and ≤ 0.08 indicates acceptable model fit. Both CFI and TLI should have a value of ≥ 0.95 for acceptable and a value of ≥ 0.97 for good model fit. For comparing fit between a specified and a nested model, the option DIFFTEST provided by Mplus was applied to account for the WLSMV estimation method [30].

The calculation of Cronbach’s alpha is based on the covariance matrix of metric items, whereas for ordinal data, the polychoric correlation matrix is required. The latter serves as the basis for calculating ordinal alpha (α_pol_) for each WAI factor in the final model in this study. This approach has been shown to be more accurate in estimating the internal consistency than Cronbach’s alpha, avoiding underestimation [26]. To allow for comparability with previous studies, in this study the traditional Cronbach’s Alpha (α) is reported as well. Both, α_pol_ and α can be interpreted as the lower bound of reliability.

## Results

The sample consists of n = 4511 respondents. From these, 310 respondents with not at least a marginal or irregular employment relationship were excluded from analyses as well as 74 self-employed and 159 other respondents who were not subject to social security contributions. Data from n = 3968 respondents (51% male) was retained for statistical analyses. Table 2 gives an overview of the sociodemographic data of the sample.

**Table 2:** Characteristics of the sample (*N* = 3968)

Variables	*n* (%)^a^
Sex	
Male	2029 (51)
Female	1939 (49)
Age groups	
31–40 years	965 (24)
41–50 years	1658 (42)
51–60 years	1345 (34)
Employment	
Full-time (≥ 35 h/week)	2879 (73)
Part-time (between 14 and 34 h/week)	945 (24)
Marginally or irregular employed	144 (4)
Vocational education	
Vocational or technical certificate/diploma from a company	2013 (51)
Vocational or technical certificate/diploma from a college	315 (8)
Bachelor degree from a vocational college	599 (15)
Master’s or professional degree from a university of applied sciences	346 (9)
Master’s or professional degree from a university	483 (12)
Other	211 (5)
Highest school degree	
Degree—grade 9 or less	1013 (26)
Degree—grade 10	1608 (41)
High school degree—grade 12/13	1278 (32)
Other/without	68 (2)
Born in Germany	
Yes	3548 (89)
No	419 (11)

^a^Deviation from the total *N* is due to missing values

For the WAI indicators, there are 12 missing data patterns, varying in frequency between 0.03 and 0.76% and the sum of all missing data patterns is < 2.5%. The most frequent missing data patterns are those which include indicators that are calculated as combined scores over more than one item and are hence more prone to missing values.

The indicators of the WAI show positive polychoric intercorrelations, ranging between *r*_pol_ = 0.22 and 0.59 (Table 1). Correlations between the WAI indicators and the traditional WAI score, calculated as an unweighted sum score over all indicators, are positive, ranging between *r*_pol_ = 0.54 and 0.76. Internal consistency of the one-factor WAI is polychoric ordinal alpha α_pol_ = 0.82, while Cronbach’s alpha based on Pearson correlations is α = 0.75.

For the three specified models A, B and C, CFAs resulted in a significant χ^2^-test (Table 3). The one-factor model A does not fit the empirical data: all fit indices CFI, TLI and RMSEA are not within an acceptable range.

**Table 3:** Results of the CFA (fit indices)

Model		*df*	χ^2^	χ^2^_diff_ Model comparison with	CFI	TLI	RMSEA	90%-CI RMSEA
A	1-factor-model	14	632.25**	–	0.92	0.88	0.11**	[0.10; 0.11]
B	2-factor-model (Items WAI3 and WAI5 loading on factor WAI_F2)	13	469.53**	A (*df* = 1) 121.57*	0.94	0.91	0.09**	[0.09; 0.10]
C	2-factor-model (Items WAI3, WAI4 and WAI5 loading on factor WAI_F2)	13	289.60**	A (*df* = 2) 232.89*	0.97	0.94	0.07**	[0.07; 0.08]

*N* = 3968

*df* degree of freedom; *χ*^*2*^ Chi-square-test; *χ*^*2*^_*diff*_ Chi-square-difference-test, *CFI* comparative-fit-index, *TLI* Tucker–Lewis index, *RMSEA* root-mean-square-error of approximation, *CI* confidence interval

*p < 0.05, **p < 0.001

In the two-factor model B, the fit indices indicate poor fit as well even though the χ^2^-difference test indicates significant improvement in model fit compared to model A. In model B, the factors are correlated with ρ = 0.73 and factor loadings range from 0.57 ≤ λ ≤ 0.78.

Finally, fit of model C—with WAI4 loading on the second factor—is acceptable with regard to the fit indices CFI and RMSEA (not acceptable for TLI) and the factors are correlated with ρ = 0.77. Compared to model A, the χ^2^-difference test indicates significant improvement in model fit of model C. Furthermore, the descriptive fit indices suggest a better fit of model C to the empirical data compared to model B. Note, however, that no difference test between models B and C is possible since the models are not nested. In Table 4, the factor loadings of the WAI indicators on the factors for all three models are shown.

**Table 4:** Factor loadings of the Work Ability Index items on the factors of the three tested models A–C

Model	A	B		C	
	WAI_g	WAI_F1	WAI_F2	WAI_F1	WAI_F2
Item	λ (*SE*)	λ (*SE*)	λ (*SE*)	λ (*SE*)	λ (*SE*)
WAI1: current work ability	0.68 (0.01)	0.69 (0.01)		0.71 (0.01)	
WAI2: work ability in relation to job demands	0.72 (0.01)	0.72 (0.01)		0.76 (0.01)	
WAI3: number of current diseases (last 12 months)	0.53 (0.02)		0.67 (0.02)		0.57 (0.02)
WAI4: estimated work impairment due to diseases	0.77 (0.01)	0.78 (0.01)			0.88 (0.01)
WAI5: sick leave (last 12 months)	0.46 (0.02)		0.57 (0.02)		0.50 (0.02)
WAI6: estimation of own work ability 2 years from now	0.63 (0.02)	0.63 (0.02)		0.64 (0.02)	0.19 (0.03)
WAI7: mental resources	0.59 (0.01)	0.60 (0.01)		0.61 (0.01)	
Factor correlation	–	0.73		0.77	

*N* = 3968

*SE* standard-error

All factor loadings (λ) are statistical significant with *p* < 0.001

Based on model C, a coefficient of internal consistency was estimated separately for each factor. For both factors, ordinal alpha (α_pol_1_ = 0.78 for WAI_F1 and α_pol_2_ = 0.69 for WAI_F2) is higher than Cronbach’s alpha (α_1_ = 0.70 for WAI_F1 and α_2_ = 0.66 for WAI_F2).

## Discussion

The aim of the study was to examine the factorial validity of the WAI within a population based random sample of employees in Germany. The results of the analyses suggest that the intercorrelations between indicators of the WAI cannot be explained by a simple one-factor structure. Thus, both the ordinal alpha as well as Cronbach’s alpha reported for a common factor cannot be reliably interpreted as a lower bound of reliability. Rather, the results imply that there are two distinguishable, albeit correlated factors underlying work ability. A model with two correlated factors (model C) fits the data best. In this model, the indicators WAI1, WAI2, WAI6 and WAI7 represent a factor for the subjective current and future work ability as well as individual resources. The second factor with indicators WAI3, WAI4 and WAI5 represents an individual health related factor.

The WAI is based on a multidimensional perspective of work ability that considers individual working conditions, mental resources and health. However, this multidimensionality is not accommodated for by the traditional index which is computed as an unweighted sum score, the latter procedure reflecting the assumption of an underlying single factor. Only one study by Bethge et al. [18] supports this assumption while other studies report diverging results regarding the psychometric properties of the WAI [17, 19–22, 24]. This is exemplified by Martus et al. [20] who established a two-factor model like model C in this study and additionally a model with double-loadings of WAI4 and WAI6 on both factors. However, such double-loadings undermine an easy interpretation of the factors. We have also examined this model with double-loadings in our analyses (results not shown here): This model has better model fit; however, that comes with the price of lower parsimony and lower interpretability. The more restrictive model—our model C—solely allowing single loadings, has acceptable model fit and can be interpreted more easily. A two-factor model consistent with model C in the present study has been established by Radkiewicz and Widerszal-Bazyl [22] via principal component analyses on data of a sample of European nurses in seven out of nine countries. It is noteworthy that the results of the present study are similar to those of Radkiewicz and Widerszal-Bazyl [22] who have used a single profession sample (nurses) and a different kind of statistical analyses.

In some previous studies, models with three correlated factors have been established [17, 19, 21]. However, these analyses were based on the 10 individual items of the WAI and applied exploratory factor analysis. Since we have restricted ourselves to the seven WAI1 to WAI7 indicators for our analyses, it was not possible to identify a three factor model with CFA.

### Strengths and Limitations

A strength of the present study in contrast to previous studies [17–22, 24] is that it is based on a random sample of employees who are subject to social security contributions in Germany. Therefore, the sampling frame is clearly defined and the interpretation of results is not hampered by a selection bias [27] as would be expected for studies using ad hoc samples or single professional groups. Nevertheless, the exclusion of freelancers, self-employed and civil servants as well as the restriction to the age group of 31–60 year old participants can be seen as a weakness of the study because no statements can be made about the structure of the WAI for groups who are not within the sampling frame. Thus, the results of the study are generalizable to all employees subject to social security contributions in Germany aged 31–60 years, which represented the target population for the analyses, while a generalization to civil servants, freelancers and self-employed as well as to those younger than 31 and older than 60 years is not possible. This should be considered when applying the WAI. The factor structure in younger or older samples or occupational groups other than those in this study may be different.

Another strength of this study is the usage of software and routines appropriate for the scaling properties of the WAI indicators: The assumption of a metric measurement scale does not hold true for all indicators of the WAI. Instead of a covariance matrix based on Pearson correlations, the polychoric correlation matrix of the indicators was the starting point for model estimation to avoid underestimation of the correlations between the variables. Due to the lack of multivariate normal distribution and the ordinal metric of WAI3 to WAI7, a robust estimation method (WLSMV) was applied for CFA. In studies comparing the performance of ML estimation versus WLSMV estimation, WLSMV was less biased in estimating the factor loadings, while the ML estimator underestimated the factor loadings and there was a tendency that correct models were falsely rejected [31–33]. Because no details are given in the studies applying principal component analysis [17, 21, 22, 24] it is not possible to say whether a bias occurred and if so, in which direction.

The estimation of an ordinal alpha as a lower bound of the reliability, based on the polychoric correlation matrix, represents a further strength of the study. As shown, two correlated factors are underlying the WAI, a result reported by some previous studies as well [20, 22, 24]. Cronbach’s alpha for the whole WAI cannot be interpreted in a meaningful way when there is evidence for more than one underlying factor as has been done before [17–22]. Therefore, for correct interpretation, internal consistency has to be calculated for each individual factor. Our analyses show that for both factors ordinal α_pol_ is higher than Cronbachs’s alpha. This was to be expected because with the Pearson correlation matrix, the relationships between the indicators are underestimated compared to the polychoric correlation matrix [26]. The fact that Cronbach’s alpha for the two factors in our analyses is partly lower compared to Cronbach’s alpha based on a common factor for all indicators combined—as has been done in previous studies [17, 19–22]—was to be expected as well [37]. Nevertheless, the values for Cronbach’s Alpha in our study—given the brevity (e.g., only three indicators for factor 2)—are acceptable. A calculation based on assumptions that do not hold true and a possible overestimation of the alpha values would strongly affect the interpretation of individual scores when using the WAI as a diagnostic tool: It would wrongly imply high precision of individual test scores.

Due to the data protection requirements for the scientific use file, it is not possible to present information on the different types of occupations held by study participants which can be considered a limitation of the study. However, interested readers may refer to the descriptive presentation of occupational clusters in the cohort profile [27]. The participants in S-MGA were employed on the time of sampling. The exclusion of non-employed or early retired individuals or homemakers in S-MGA is an important difference to other population-based studies. It can be assumed that this introduces a bias towards participants with less impairments and a higher level of functioning who are probably healthier than those of the overall population. However, this should not be considered a limitation because the sampling frame of S-MGA is closer to the working population than to the overall population.

### Implications

The two-factor structure of the WAI as presented in this study has implications for the WAI’s application in occupational medicine: Although the theory behind the WAI considers work ability to be multifactorial—including the current and future work ability in relation to physical and mental work demands, health and individual resources—in practice and research the index has always been calculated as a simple sum score. The empirical results do not support the approach of using a single index for the assessment of work ability as has been done in the past. Instead, when interpreting the WAI one should take into account its two-factor structure to avoid incorrect conclusions, both in terms of case-by-case diagnostic in practice and population analysis in research. Future research should examine the applicability of calculating two index scores based on the two factors. These should be weighted according to the factor loadings from the structural equation model. Furthermore, information on whether the two scores have differential predictive validity with regard to relevant outcome measures, e.g. sick leave or early retirement, is not available as of yet and has to be gained in order to properly apply the two values based on the factors in a practical context. It is possible that one factor has a stronger predictive value for relevant outcome measures, rendering a combined score a weaker predictor. For example, it has been shown that the positive expectation of one’s own return to work has a strong effect on successful return to work [15, 16]. The similarity of “positive expectations” to indicator WAI6 (own prognosis of work ability 2 years from now) and the latter loading on the factor for subjectively estimated work ability, suggest that this first factor with its subjective components could have a stronger predictive validity for successful return to work than the second factor or the sum score. Taking this thought further, the first factor might possibly be a strong predictor of early retirement. In a wider context, the subjectively estimated work ability could help to identify those at risk who might require preventive actions. These aspects warrant further research.

## Conclusion

In summary, the study has shown that the one-factor structure of the WAI as proposed by its developers is not tenable. Rather, there are two related factors underlying the instrument. The first factor represents the subjectively estimated work ability, considering working conditions and individual resources, while the second represents an individual health related factor.
